# Lack of Association between NLGN3, NLGN4, SHANK2 and SHANK3 Gene Variants and Autism Spectrum Disorder in a Chinese Population

**DOI:** 10.1371/journal.pone.0056639

**Published:** 2013-02-26

**Authors:** Yanyan Liu, Yasong Du, Wenwen Liu, Caohua Yang, Yan Liu, Hongyan Wang, Xiaohong Gong

**Affiliations:** 1 The MOE Key Laboratory of Contemporary Anthropology and State Key Laboratory of Genetic Engineering, School of Life Sciences, Fudan University, Shanghai, China; 2 Department of Child and Adolescent Psychiatry, Shanghai Mental Health Center, Shanghai, China; 3 School of Medicine, Shanghai Jiao Tong University, Shanghai, China; 4 Genesky Biotechnologies Inc., Shanghai, China; University of Illinois at Chicago, United States of America

## Abstract

Autism spectrum disorder (ASD) is a neurodevelopmental disorder characterized by deficits in social communication, absence or delay in language development, and stereotyped or repetitive behaviors. Genetic studies show that neurexin-neuroligin (NRXN-NLGN) pathway genes contribute susceptibility to ASD, which include cell adhesion molecules *NLGN3*, *NLGN4* and scaffolding proteins *SHANK2* and *SHANK3*. Neuroligin proteins play an important role in synaptic function and trans-synaptic signaling by interacting with presynaptic neurexins. Shank proteins are scaffolding molecules of excitatory synapses, which function as central organizers of the postsynaptic density. Sequence level mutations and structural variations in these genes have been identified in ASD cases, while few studies were performed in Chinese population. In this study, we examined the copy numbers of four genes *NLGN4, NLGN3, SHANK2,* and *SHANK3* in 285 ASD cases using multiplex fluorescence competitive polymerase chain reaction (PCR). We also screened the regulatory region including the promoter region and 5′/3′ untranslated regions (UTR) and the entire coding region of *NLGN4* in a cohort of 285 ASD patients and 384 controls by direct sequencing of genomic DNA using the Sanger method. DNA copy number calculation in four genes showed no deletion or duplication in our cases. No missense mutations in *NLGN4* were identified in our cohort. Association analysis of 6 common SNPs in *NLGN4* did not find significant difference between ASD cases and controls. These findings showed that these genes may not be major disease genes in Chinese ASD cases.

## Introduction

Autism spectrum disorder (ASD) is a neurodevelopmental disease with complex genetic and clinical heterogeneity. The core symptoms of ASD are impaired social interactions, deficient communication, restricted interests, and stereotyped activity patterns [1]. The male∶female ratio of 4∶1 in ASD suggests the involvement of the X chromosome in the etiology of ASD [2], [3], [4], [5]. Genetic studies show that the neurexin-neuroligin (NRXN-NLGN) pathway genes contribute susceptibility to ASD, which include cell adhesion molecules *NLGN3*, *NLGN4* (also named *NLGN4X*), *NRXN1* and scaffolding proteins *SHANK2* and *SHANK3* [6], [7], [8], [9], [10].

NLGN proteins, expressed highly in brain, are postsynaptic adhesion molecules interacting with presynaptic NRXNs in a calcium-dependent manner [11]. The cytoplasmic tails of neuroligins contain a binding site for PDZ domains of scaffolding proteins (PSD-95, S-SCAM). The X-linked *NLGN3* and *NLGN4* are the first discovered ASD-associated genes in this pathway [12]. A frame-shift mutation in esterase domain of the *NLGN4* gene was identified in two brothers, one with autism and the other one with Asperger syndrome [12]. This finding triggered the genetic and biological studies of neuroligins in ASD. About ten of the studies found non-synonymous mutations or deletions of *NLGN4* in ASD [12], [13], [14], [15], [16], [17], [18], [19], [20], while the others were negative [4], [21], [22], [23], [24]. However, only one putative causative missense mutation R451C in *NLGN3* was detected in two male ASD siblings until now [12]. Compared with the *NLGN3* gene, *NLGN4* was more frequently discovered to have ASD-related rare functional mutations in coding regions or promoter regions [13], [14], [18], [19], [20], deletions [16], [17], or common polymorphisms associated with ASD [15]. Though the mutations of *NLGN4* happen in only a small fraction of ASD cases, their impacts on synaptic function provide an important perspective for understanding the pathogenesis of ASD. Besides the coding region, the regulatory region and copy number of *NLGN4* are deserved to be studied in ASD since they may affect the expression level of *NLGN4*.

SHANK proteins contain multiple interaction sites and assemble many molecules, including guanylate kinase-associated proteins (GKAPs), which links to PSD-95 [6], [25]. Durand et al. firstly reported nonsynonymous/frameshift mutations and deletions/duplications in *SHANK3* in ASD cases [26]. Gauthier et al. identified a splice variant at amino acid 755 and confirmed the deleterious effect of this mutation by RT-PCR [27]. Shank3 mutant mice display autistic-like behaviors and have severely synaptic dysfunction [28], [29]. Interestingly, recent two studies have identified copy number variations (CNVs) and rare mutations in *SHANK2* in ASD subjects, suggesting the implication of *SHANK2* in ASD [9], [10].

In this study, we sequenced the regulatory region including the promoter region and 5′/3′ untranslated regions (UTR) and the entire coding region of *NLGN4* in a cohort of 285 ASD patients and 384 controls by direct sequencing of genomic DNA using the Sanger method. We also examined the CNVs of *NLGN4, NLGN3, SHANK2* and *SHANK3* in 285 ASD cases using multiplex fluorescence competitive polymerase chain reaction (PCR). Moreover, six common single nucleotide polymorphisms (SNPs) within *NLGN4* were identified and analyzed for the association with ASD.

## Materials and Methods

### Ethics Statement

The study was approved by the Ethics Committee of Shanghai Mental Health Center and the Ethics Committee of the School of Life Sciences, Fudan University. Informed, written consent was obtained from the controls, the parents or guardians of the children.

### Subjects

A total of 285 patients with ASD were recruited from Department of Child and Adolescent Psychiatry, Shanghai Mental Health Center, China. All probands met DSM-IV diagnostic criteria for autism, Asperger syndrome or pervasive developmental disorder not otherwise specified (PDD-NOS). Cases were excluded if they had known medical disorders or chromosomal abnormalities. The patients were 246 males and 39 females with a mean age of 7.05 (SD = 3.36 years). All individuals were Han Chinese.

384 unrelated ethnically-matched healthy controls were recruited from Fudan University. There were 311 males and 73 females.

### DNA Sequencing

The reference sequence of *NLGN4* gene was acquired from the UCSC Genome Browser (NM_181332). The promoter region (2000 bp upstream of the transcription start site), six exons including the 5′/3′ UTRs and the open reading frame (ORF), and ten exon-intron junctions were sequenced in 285 ASD patients and 384 controls. Totally, 9110 basepair (bp) of untranslated region and 2451 bp of coding region in *NLGN4* were sequenced. Genomic DNA was isolated from whole blood using Puregene Blood Core Kit C (Qiagen, Valencia, CA). PCR primers were designed by PRIMER 3 (http://frodo.wi.mit.edu/primer3/). The information of primers is shown in Table S1. The pairs of primers for exons 3, 5, and 6 are specific for *NLGN4*, while those for exons 1, 2, and 4 can amplify the sequences of *NLGN4* and *NLGN4Y* simultaneously due to sequence homology. PCR was performed in a 10 µl reaction mixture containing 1 µl 10×buffer (Mg^2+^ Plus), 1 µl dNTP mixture (2.5 mM), 0.2 µl primers (10 µM), 0.05 µl Takara Hotstar Taq (5 U/µl), and 1 µl genomic DNA (20 ng/µl). Thermo cycling conditions consisted of 35 cycles with an initial denaturation at 95°C for 5 min, followed by denaturation at 95°C for 30 s, annealing at 58°C for 30 s, extension at 72°C for 30 s, and a final extension at 72°C for 5 min. We carried out all sequencing reaction by BigDye Terminator version 3.1 in ABI 3100 sequencers (Applied Biosystems, Foster City, CA).

### Detection of Copy Number

The copy numbers of four genes (*NLGN4, NLGN3, SHANK2,* and *SHANK3*) were measured by a custom-by-design Multiplex AccuCopy™ Kit (Genesky Biotechnologies Inc., Shanghai, China) based on a multiplex fluorescence competitive PCR principle as described by Du et al. [30]. The reference genome sequences were obtained from the UCSC Genome Browser (hg19; http://genome.ucsc.edu). Three reference genes (*POP1*, *RPP14*, and *TBX15*) were utilized for normalization. Nine target genomic segments within the four genes (two or three segments for each gene) were chosen for the AccuCopy assay. The forward and reverse primers of target segments were provided in Table S2. Our data were produced according to the manufacturer's manual. Briefly, a 20 µl PCR reaction was prepared for each sample, containing 1× AccuCopy™ PCR Master Mix, 1× Fluorescence Primer Mix, 1× Competitive DNA mix, and ∼10 ng sample DNA. The PCR program was described as followed: 95°C 10 min; 11 cycles of 94°C 20 s, 65°C −0.5°C/cycle 40 s, 72°C 1.5 min; 24 cycles of 94°C 20 s, 59°C 30 s, 72°C 1.5 min; 60°C 60 min. PCR products were diluted 20-fold before loaded on ABI3730XL sequencer. Raw data were analyzed by GeneMapper4.0 and height/area data for all specific peaks were exported into an excel file. The sample/competitive (S/C) peak ratio was calculated for the target segments and three reference genes (*POP1, RPP14,* and *TBX15*) and the S/C ratio for each target fragment was first normalized to three reference genes respectively. The three normalized S/C ratios were further normalized to the median value in all samples for each reference gene respectively and then averaged. If one of the three normalized S/C ratios deviated more than 25% from the average of the other two, it was excluded for further analysis.

### Statistics

The association analysis of 6 common SNPs was performed between cases and controls using the chi-square test in SPSS15.0. Linkage disequilibrium (LD) analysis of SNPs and the haplotype association were analyzed using Haploview 4.2 software. LD between the SNPs was measured by a pairwise D′ statistic. The structure of the LD block was examined using the method of Gabriel et al. [31]. Haplotype frequencies were estimated using an accelerated expectation-maximization algorithm method [32]. Haplotype frequencies occurring at <1% were excluded from the analysis. The haplotype association test was performed using the chi-square test. The significance level for all statistical tests was 0.05.

## Results

**Figure 1:**
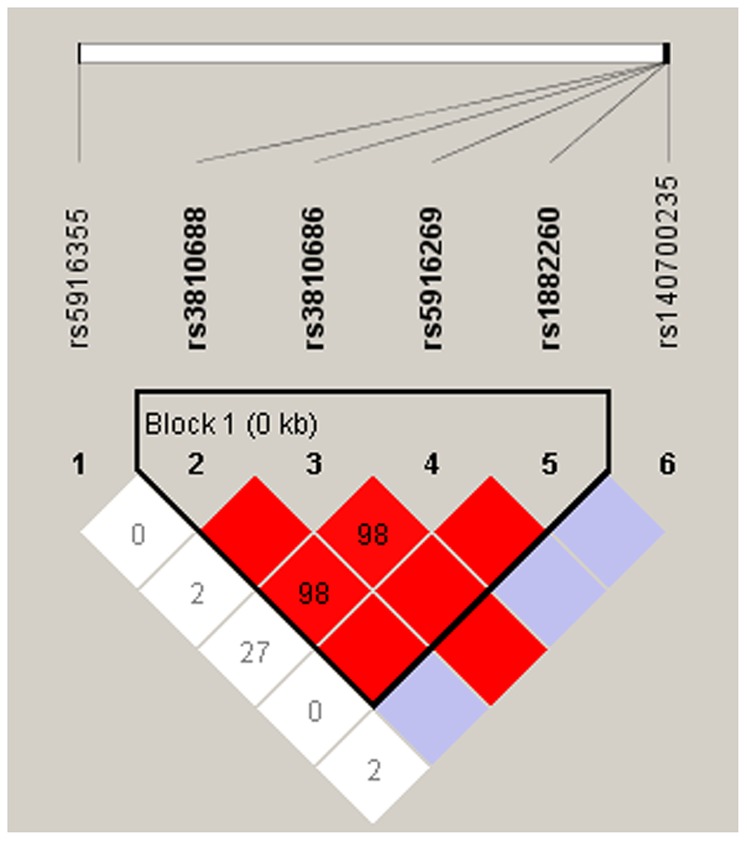
Linkage disequilibrium block of *NLGN4* gene. The color of each square from light to dark represents the level of LD from low to high. White: complete recombination; blue: partial linkage; red: complete linkage.

Only one synonymous mutation rs7049300 (Thr311Thr) was found in one ASD individual. No non-synonymous mutation in *NLGN4* was detected in our cohort. Direct sequencing of the coding region and regulatory region of *NLGN4* identified six common SNPs (minor allele frequency >5%). They were rs5916355 in the promoter region, rs3810688, rs3810686, rs5916269, rs1882260, and rs140700235 in 3′UTR. Five percent of samples were selected randomly for validation by sequencing in the opposite direction. No genotyping error was detected. Association analysis of these six SNPs with ASD did not show significant difference of allele frequencies between ASD patients and controls (Table 1). The LD block of 6 SNPs were constructed (Figure 1). The polymorphisms rs3810688, rs3810686, rs5916269, and rs1882260 were in strong LD with each other and therefore formed a haplotype block. Four common haplotypes with the frequency >1% were inferred. They were GACT (0.659), GGCC (0.172), AGTT (0.132), and AGCT (0.031). Haplotype analysis did not find significant frequency difference between cases and controls.

**Table 1:** The distribution of allele frequencies for 6 SNPs in *NLGN4* gene.

Position	SNP	Allele frequency	Allele *P*-value
promoter	rs5916355	T/G	0.689
	case	288(0.94)/20(0.06)	
	control	344(0.94)/21(0.06)	
3′UTR	rs3810688	G/A	0.489
	case	258(0.83)/54(0.17)	
	control	357(0.85)/65(0.15)	
3′UTR	rs3810686	G/A	0.975
	case	105(0.34)/206(0.66)	
	control	142(0.34)/280(0.66)	
3′UTR	rs5916269	C/T	0.991
	case	244(0.87)/36(0.13)	
	control	365(0.87)/54(0.13)	
3′UTR	rs1882260	C/T	0.706
	case	48(0.17)/237(0.83)	
	control	75(0.18)/343(0.82)	
3′UTR	rs140700235	A/G	0.478
	case	275(0.96)/11(0.04)	
	control	405(0.97)/12(0.03)	

*P value not corrected for the multiple testing.

**Figure 2:**
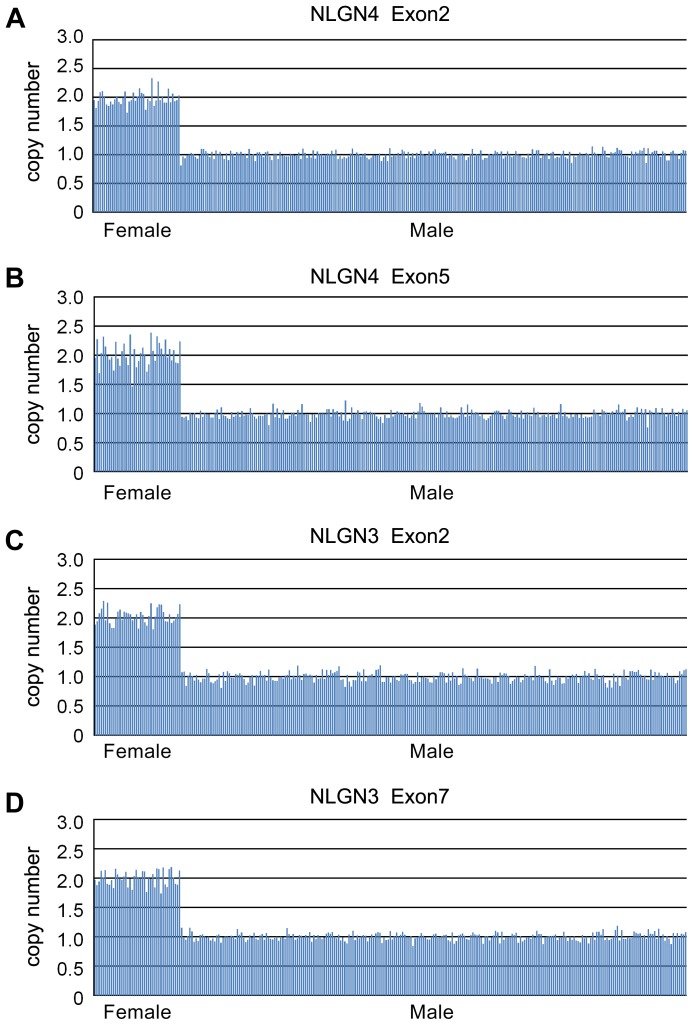
Assays of copy number in X-linked *NLGN4* and *NLGN3* genes. The copy number states of two segments for each gene for each ASD patient were shown in two panels. Panel A: exon2 of *NLGN4*; Panel B: exon5 of *NLGN4;* Panel C: exon2 of *NLGN3*; Panel D: exon7 of *NLGN3*. Each column indicates a patient. All female ASD cases showed two copy of *NLGN4/NLGN3* and male ASD cases had one copy of *NLGN4/NLGN3*.

**Figure 3:**
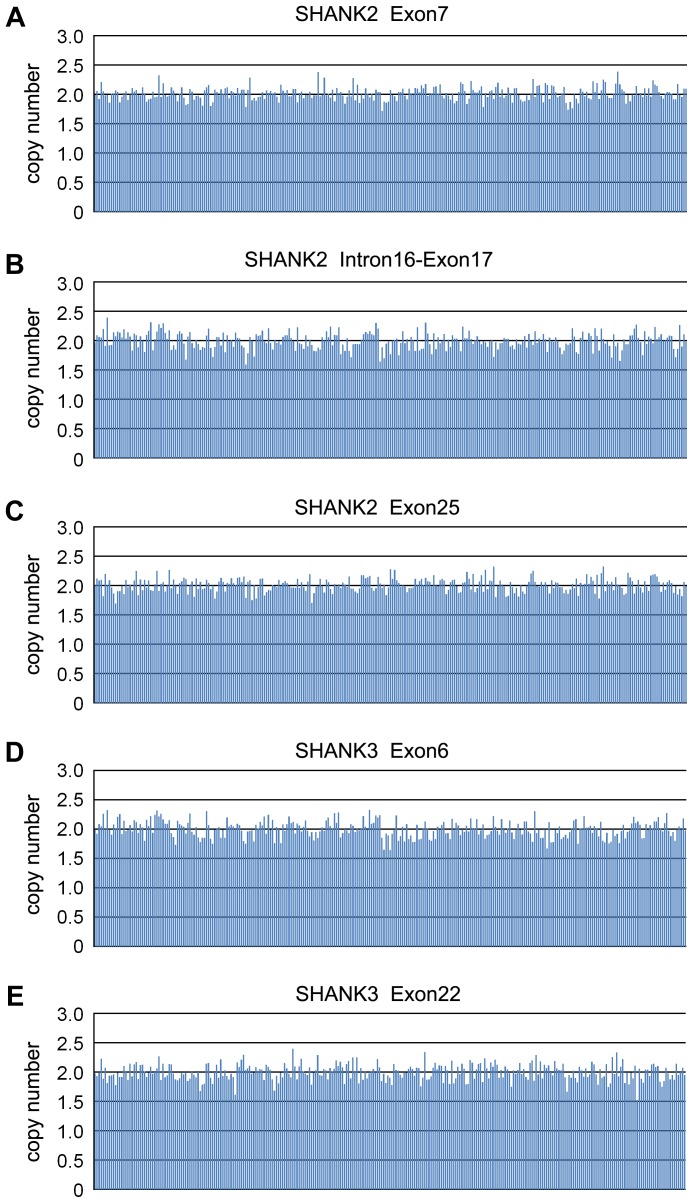
Assays of copy number in *SHANK2* and *SHANK3* genes. The copy number states of five segments in ASD patients were shown in five panels. Panel A: exon7 of *SHANK2*; Panel B: intron16-exon17 of *SHANK2*. Panel C: exon25 of *SHANK2*; Panel D: exon6 of *SHANK3*; Panel E: exon22 of *SHANK3*. Each column indicates a patient. All ASD cases showed two copy of *SHANK2/SHANK3.*

The calculated copy numbers of both two fragments of *NLGN4* ranged between 0.76 and 1.22 in 246 male samples, and ranged between 1.69 and 2.38 in 39 female samples, which indicated unchanged copy number of *NLGN4* for all samples (Figure 2). Neither CNVs of X-linked *NLGN3* were detected in our cohort (Figure 2). The calculated copy numbers of five segments in *SHANK2* and *SHANK3* ranged between 1.52 and 2.40 in 285 ASD cases, indicating unchanged copy number (Figure 3).

## Discussion

Since the first report of *NLGN4* mutations in ASD was published in Nature Genetics by Jamain et al. in 2003, more than ten studies investigated the relationship between *NLGN4* genetic variants and ASD [4], [12], [13], [14], [15], [16], [17], [18], [19], [20], [21], [22], [23], [24]. A 2-base-pair deletion of *NLGN4*, leading to a premature stop codon in the middle of the sequence, cosegregated with the affected members with mental retardation and/or autism in a large French family [13]. A deletion of exons 4–6 of *NLGN4* was found in a family with a wide variation in neuropsychiatric illness including autism, Tourette syndrome, attention deficit hyperactivity disorder, learning disorder, anxiety, and depression [16]. Daoud et al. studied 96 ASD patients and identified a novel mutation (−335G>A) in the promoter region of *NLGN4* in a patient with autism and mental retardation [18]. This mutation was associated with the increased expression level of NLGN4 in the patient, supported by the functional study *in vitro*. An amino-acid substitution in *NLGN4* (R87W) was identified in two brothers with ASD [20]. This mutation impaired glycosylation procession of NLGN4, affected the location of NLGN4 to the cell surface, and inactivated the synapse-formation activity of NLGN4. The Nlgn4 knockout mice displayed highly selective deficits in reciprocal social interactions and communications that are the key symptoms of ASD in human [33].

Only one study investigated the susceptibility of *NLGN3* and *NLGN4* to ASD in Chinese population so far [24]. Seven known ASDs-related rare mutations in *NLGN3* and *NLGN4* genes were screened and twelve intronic SNPs were genotyped for case-control association analysis in 229 ASDs cases and 184 controls in a Chinese cohort [24]. A common SNP rs4844285 in *NLGN3* gene was associated with ASD (SNP allele: P = 0.048). Six SNPs in the introns of *NLGN4*, different with the ones examined in our study, were analyzed and showed no frequency difference between ASD cases and controls [24]. In the present study, we investigated the role of *NLGN4* in 285 Chinese ASD cases by comprehensively testing genetic variants including rare mutations in the coding region and regulatory region, copy number variations and common SNPs. No positive results were identified, suggesting *NLGN4* gene is not a major susceptibility gene in our ASD cohort.

To investigate the possibility of other genes in neurexin-neuroligin pathway involved in the etiology of ASD, we examined the copy number of *NLGN3, SHANK2,* and *SHANK3* in this cohort. Shank proteins are scaffolding molecules of excitatory synapses, which function as central organizers of the postsynaptic density and are believed to play an important role in the formation and maturation of dendritic spines. Rare *de novo* CNVs and nonsense/frameshift mutations in *SHANK2* and *SHANK3* have been identified in ASD and mental retardation cases [9], [10], [26], [27], [34]. Berkel et al. performed a genome-wide microarray scan for CNVs and sequenced the whole exons of *SHANK2* in a German cohort of 184 mental retardation cases and a Canadian cohort of 396 ASD probands [9]. Two *de novo* deletions and a *de novo* nonsense mutation of *SHANK2* were identified in unrelated mental retardation and ASD individuals. Haploinsufficiency of *SHANK3* is associated with ASD and mental retardation in human patients and results in altered synaptic plasticity and reduced social behaviors in mice [28]. In the present study, we found no deletions or duplications in *NLGN3, SHANK2* and *SHANK3*, indicating the limited role of these genes in Chinese ASD patients. Considering that we did not screen these genes for sequence level variants and did not investigate other genes in this pathway like *NRXN3* and *SHANK1*, recently reported to be associated with ASD [35], [36], the pathogenic role of neurexin-neuroligin pathway genes in ASD are warranted to study.

One limitation of this study should be considered. Nine missense/nonsense mutations and 3 deletions within *NLGN4* or *NLGN3* have been reported by different studies involving about 1500 ASD cases, indicating a low frequency (no more than 1%) of rare variants within *NLGN3* and *NLGN4* in ASD [4], [12], [13], [14], [15], [16], [17], [18], [19], [20], [21], [22], [23], [24]. Based on these data, our sample size was relatively small to find rare variants. However, it was the first time to screen the regulatory region and the entire coding region of *NLGN4* as well as examine the copy numbers of four genes *NLGN4, NLGN3, SHANK2,* and *SHANK3* in Chinese population. Though the results were negative, this study would extend our understanding about the role of these genes in ASD in different populations.

In this study, we used the multiplex fluorescence competitive PCR for copy number measurement. This newly developed method has the advantages of multiplex measurement and low measurement variation [30]. The limitation of this method is that a flase positive deletion signal will be obtained if some rare mutations happen to be inside PCR primer regions. In case that it happens, more than one fragment in target genes had better be measured, and the sequencing of primer regions will be necessary to check whether an unknown mutation is located within the primer regions if only one fragment is found to be deleted. We assayed nine fragments in four targed genes. Due to no deletion or duplication detected, we did not perform quantitative PCR validation or sequence the primer regions. Another limitation of this method was that partial deletions/duplications of targeted genes (for example, deletions of one or two exons) could be missed considering only two or three segments of genes were tested in this study.

Most previously reported studies focused on the mutations in the coding region. In our study, we also pay attention to the genetic variants which may influence the expression level of NLGN4. Six common SNPs in the promoter region and 3′ UTR of *NLGN4* were identified and studied for the relationship with ASD in our cohort. These SNPs might affect the expression of NLGN4 by binding with transcription factors or microRNA. The SNP rs5916355 in the promoter region of *NLGN4* was predicted to be a binding site for transcription factor HFH-2 using the online tool TFSEARCH. Two websites (http://www.microrna.org/microrna/getGeneForm.do and http://www.mirbase.org/search.shtml) were used to predict miRNAs which bind to the 3′UTR sequence of *NLGN4* containing five tested SNPs. The variants rs3810688, rs5916269 and rs140700235 were found located within the potential binding sites of has-miR-1293, has-miR-1324 and has-miR-5011-5p, respectively. These SNPs may affect NLGN4 expression by altering the interaction of miRNAs and mRNA. For example, according to the algorithm RNAhybrid (http://bibiserv.techfak.uni-bielefeld.de/rnahybrid/), the allele A of rs3810688 in the NLGN4 3′UTR has a mismatch with miR-1293 seed region and an altered minimum free energy value (mfe = −23.1 kcal/mol), compared with the allele G (mfe = −29.3 kcal/mol). Though we did not find any SNP which had significant frequency difference between ASD cases and controls, the validation in more samples and functional study would help us to understand the potential role of these polymorphisms in ASD.

In this study, we investigated the role of *NLGN4* in ASD by comprehensive screening of the coding region and regulatory region of *NLGN4* for mutations and examining the copy number variations of *NLGN4*. 285 ASD patients were studied and no missense mutations or deletion/duplication were identified. There was no association between 6 common SNPs in *NLGN4* and ASD. These findings showed that *NLGN4* may not play an important role in our ASD cohort.

## Supporting Information

Table S1 **The primer and PCR condition of** ***NLGN4*** **gene.**
(DOC)

Table S2 **The primers of four genes for multiplex competitive PCR.**
(DOC)
