# Supplementary material for: Lack of Association between NLGN3, NLGN4, SHANK2 and SHANK3 Gene Variants and Autism Spectrum Disorder in a Chinese Population
Source: PLoS One. 2013 Feb 26;8(2):e56639. doi: 10.1371/journal.pone.0056639 (PMC3582503; doi:10.1371/journal.pone.0056639)
Supplement: Table S1 — The primer and PCR condition of NLGN4 gene. (DOC) [file pone.0056639.s001.doc]

**Table S1. The primer and PCR condition of *NLGN4* gene**

|  | **Position** | **Forward primer/Reverse primer** | **Size** | **Condition (Tm)** |
| --- | --- | --- | --- | --- |
| NLGN4-PCR1 | Promoter | tccttgtgcacaccttcttg/tcctgtggatttcttgtgga | 595 bp | 60℃ |
| NLGN4-PCR2 | Promoter | cctatcttgttggtaaaaggtgct/cgaggacgggggtatgac | 685 bp | 60℃ |
| NLGN4-PCR3 | Promoter | gcatcttgaacccaagcaat/ctcgggagatagagcaggtg | 604 bp | 60℃ |
| NLGN4-PCR4 | Exon1 | aaggtttgctgtacgcgtct/gtgaggctttccatcctttg | 699 bp | 60℃ |
| NLGN4-PCR5 | Exon2 | ggtgatgttcttgctgttgc/actgtgcttggctgtcaatg | 609 bp | 60℃ |
| NLGN4-PCR6 | Exon2 | gacagctgtggatgtggatg/tgcacaagaggtattgttttctg | 545 bp | 60℃ |
| NLGN4-PCR7 | Exon3 | gggaaatcagtgaatctccttt/tctgcattggtttgagaagg | 454 bp | 58℃ |
| NLGN4-PCR8 | Exon4 | tctaattggtgggggtgaga/gggtgacagagcaaatggaa | 507 bp | 58℃ |
| NLGN4-PCR9 | Exon5 | ccagaaactcacatgcctga/aaggttgtccacgaagttgg | 515 bp | 58℃ |
| NLGN4-PCR10 | Exon5 | ggcgagttcctcaactacga/accccaacacgaagatgaac | 548bp | 58℃ |
| NLGN4-PCR11 | Exon6 | tgctgcttgggtattttgtg/ggattgttggcaggagtgat | 477 bp | 58℃ |
| NLGN4-PCR12 | Exon6 | atcactcctgccaacaatcc/gaagggaaatagggcaaagc | 550 bp | 58℃ |
| NLGN4-PCR13 | 3’ UTR | caccatcaccatgattccaa/gtgctgggcaaaatctcatt | 604 bp | 60℃ |
| NLGN4-PCR14 | 3’ UTR | aactcagccaaggacacttga/tttggggacccatctctgta | 699 bp | 60℃ |
| NLGN4-PCR15 | 3’ UTR | gacggtctgtgtggcctatt/gaaaacccatgcaaagcagt | 631 bp | 60℃ |
| NLGN4-PCR16 | 3’ UTR | tggatttgtgtatccggtca/aatgttgcttcacgtgtgct | 612 bp | 60℃ |
| NLGN4-PCR17 | 3’ UTR | ggctccattgtgggttattc/caaatgtcacaagcctgacg | 574 bp | 60℃ |
| NLGN4-PCR18 | 3’ UTR | ccatgttggtttcgtctgtc/aaaacacgccacctaccaag | 562 bp | 60℃ |
