# Supplementary material for: Lack of Association between NLGN3, NLGN4, SHANK2 and SHANK3 Gene Variants and Autism Spectrum Disorder in a Chinese Population
Source: PLoS One. 2013 Feb 26;8(2):e56639. doi: 10.1371/journal.pone.0056639 (PMC3582503; doi:10.1371/journal.pone.0056639)
Supplement: Table S2 — The primers of four genes for multiplex competitive PCR. (DOC) [file pone.0056639.s002.doc]

**Table S2. The primers of four genes for multiplex competitive PCR**

| **Gene** | **Source** | **Segment** | **Position** | **Forward primer/ Reverse primer** |
| --- | --- | --- | --- | --- |
| NLGN3 | NM_181303 | S1 | Exon2 | CTGAGGGAGTCCCCTTTCTGAAG/ GTGACCCTGGAGTCTGCCTCTC |
|  |  | S2 | Exon7 | AGCCATCATCCAAAGTGGCTCT/ ACACCGTGGATATGGTGGACTGT |
| NLGN4 | NM_181332 | S3 | Exon2 | GCTTCCTTGGAGCATTGCAGTT/ CTACTGCTCCCTGGAAAGCCCT |
|  |  | S4 | Exon5 | CCATCATTCAGAGCGGCACC/ AGAATGCCTGCGGAACAAGAAC |
| SHANK2 | NM_012309 | S5 | Exon7 | GCTTGGTGCATCCCCAGATTAT/ CCTACTGCTGCGAGCTTCTCCT |
|  |  | S6 | Intron16-Exon17 | TGCTTGTCTTCCCAGCTGACAC/ GGACTAAGGACCGGGGACTTCT |
|  |  | S7 | Exon25 | CAGGAGAGAGCTGGACCGCTAC/ AAAACCCATACTCAGAGGTGGGG |
| SHANK3 | NM_001080420 | S8 | Exon6 | CTGGGGGCTTCACCTGACTACA/ CTGTGAGCTGCTTCTCCACGAC |
|  |  | S9 | Exon22 | ACCTGGAGACCACAAGCACCAT/ AGAGCGGGGAACTCACTGACAC |
